# The effect of azithromycin on sputum inflammatory markers in bronchiectasis

**DOI:** 10.1186/s12890-023-02444-1

**Published:** 2023-04-29

**Authors:** L. C. Terpstra, J. Altenburg, H. J. Doodeman, Y. S. Sabogal Piñeros, R. Lutter, H. G. M. Heijerman, W. G. Boersma

**Affiliations:** 1 Department of Pulmonary Diseases, Northwest Clinics, Wilhelminalaan 12, 1812 JD Alkmaar, The Netherlands; 2 Department of Pulmonary Diseases, Amsterdam UMC, University of Amsterdam, Amsterdam, The Netherlands; 3 Department of Northwest Academy, Northwest Clinics, Alkmaar, The Netherlands; 4 Department of Experimental Immunology, Amsterdam UMC, University of Amsterdam, Amsterdam, The Netherlands; 5 Department of Pulmonary Diseases, University Medical Centre Utrecht and Utrecht University, Utrecht, The Netherlands

**Keywords:** Bronchiectasis, Azithromycin (AZM), Inflammatory markers, Inflammation

## Abstract

**Background:**

Long term macrolide treatment has been found beneficial in bronchiectasis (BE) -pathogical bronchial dilatation- possibly due to a combined anti-bacterial and immunomodulatory effect. The exact mechanism of inflammatory response is unknown. Here, we investigated the effect of maintenance macrolide treatment on the inflammatory response in BE. In addition, we assessed the inflammatory profile in BE in relation to disease severity.

**Methods:**

During the BAT randomized controlled trial (investigating the effect of 1 year of azithromycin (AZM) in 83 BE patients), data on BE severity, lung function and sputum microbiology was collected. For the current study, a wide range of inflammatory markers were analysed in 3- monthly sputum samples in all participants.

**Results:**

At baseline, marked neutrophilic but also eosinophilic inflammation was present in both groups, which remained stable throughout the study and was not affected by AZM treatment. Significant upregulation of pro-inflammatory markers correlated with FEV_1_ < 50% (TNFα, ECP, IL-21, IL-1, *p* = 0.01- 0.05), *H. influenzae (HI)* colonization (MPO, ECP, MIP-1, TNFα, IL-21, Il-8, IL-1, IL-1α, *p* < 0.001 – 0.04) and number of exacerbations (MPO, ECP, VEGF, MMP-9, *p* = 0.003 – 0.01). Surprisingly, colonization with *P. aeruginosa (PA)* was found to correlate with an attenuated inflammatory response compared to non-*PA* colonized. In placebo-treated patients, presence of an infectious exacerbation was reflected by a significant excessive increase in inflammation as compared to a non-significant upregulation in the AZM-treated patients.

**Conclusion:**

One year of AZM treatment did not result in attenuation of the inflammatory response in BE. Increasing disease severity and the presence of an exacerbation were reflected by upregulation of pro-inflammatory markers**.**

**Supplementary Information:**

The online version contains supplementary material available at 10.1186/s12890-023-02444-1.

## Introduction

Non-cystic fibrosis bronchiectasis (hereafter referred to as ‘bronchiectasis’) is characterized by a vicious cycle of bacterial colonization, airway inflammation and airway structural damage, resulting in bronchial dilatation, with recurrent infections, chronic symptoms of cough and sputum production, and an increase in severity of the disease [1–3]. The pathogenesis is poorly understood, but airway neutrophil dysfunction is considered a key component of this vicious circle of lung damage, and might result from a combination of host-derived mediators, bacterial virulence factors, and changes induced by incomplete attempts to clear biofilm-shielded bacteria [4–6].

Previous studies revealed elevated levels of several pro-inflammatory, neutrophil driven cytokines, even in stable bronchiectasis airway secretions [4, 7]. A few studies investigated the association between sputum inflammatory products and severity of the disease. Neutrophil elastase (NE) was proposed as a biomarker for exacerbations and lung function decline, and IL-8 and IL-13 were correlated with measurements of disease severity [8, 9]. Also a heterogeneity of systemic inflammation was found in bronchiectasis, with higher levels of CRP, IL-6 and plasma fibrinogen during an exacerbation [10]. Plasma fibrinogen was also associated with the severity of bronchiectasis, a worse health status and with *Pseudomonas aeruginosa (PA)*colonization [10, 11]. In addition, Neutrophil extracellular trap (NET) formations were recently identified as a key marker of disease severity and treatment response in bronchiectasis [12].

For the frequent exacerbating bronchiectasis patients, macrolide maintenance treatment has shown favourable results and is nowadays part of the standard treatment in patients with bronchiectasis [13–16]. The benefits of macrolides are believed to be based on both the antimicrobial effect and the immunomodulatory effects. The mechanisms underlying this dual effect are not completely understood but are thought to be part attributable to an anti-neutrophilic mode of action as depicted by lower levels of neutrophils chemo-attractants in sputum of bronchiectasis patients after macrolide treatment [17, 18].

In the present study we investigated the relation between the inflammatory profile in spontaneous sputum samples and the severity of bronchiectasis. In addition, we studied the inflammatory effect of azithromycin (AZM) treatment om airway inflammation markers during maintenance treatment and during an exacerbation, and we explored if higher levels of particular inflammatory markers at baseline may be predictive of an enhanced effect of AZM maintenance treatment with respect to number of exacerbations, quality of life and lung function.

## Materials and methods

### Participants

The Bronchiectasis and Long-term Azithromycin Treatment (BAT) randomized controlled trial, was a multicentre, placebo-controlled trial conducted at 14 sites in the Netherlands from 2008–2010 (Clinicaltrials.gov, number NCT00415350; Ethical approval METC Noord Holland: M07-002, CCMO: NL16025.094.07); detailed study protocols and results are provided elsewhere [13]. Participants were eligible for randomization if they had radiologically confirmed bronchiectasis, with three or more lower respiratory tract infections treated with antibiotics in the preceding year, and at least one positive sputum culture for bacterial respiratory pathogens. Patients were randomized to receive either AZM (250 mg OD) or placebo for 12 months and underwent a follow up every 3 months at the outpatient ward.

### Sputum cultures and immunological analysis

Sputum samples were collected at start of the treatment period till the end of the study at three-month intervals, and after the run-out period of 3 months. This spontaneously expectorated sputum used in the present analysis was frozen and stored at -80 °C till processing [19]. Due to the beneficial effect of AZM some patients did not expectorate sputum anymore at the end of the study period.

In short, frozen samples were quickly thawed, the volume of the sputum was set equal to its weight and 10 mM dithiothreitol (DTT) solution was added in a 1:1 ratio, followed by incubation. When not all mucus was liquified, the same volume of DDT was added again, followed by another incubation step till all mucus was liquified. After mucus was liquefied, we often found cell aggregates which were dispersed by incubation with DNase (Sigma D-5025; 150,000 U). This step was repeated when necessary. Finally, the processed samples were centrifuged after which supernatant was collected and aliquoted. As DTT reduces sulphur bridges that may affect antigenic epitopes and antibodies, we diluted samples at least 50-fold to minimize the effect of DTT as was confirmed by further serial dilutions. Additional information about the assays and sputum analysis is shown in supplemental 3. A separate sputum sample was also collected for bacteriology.

### Lung function, quality of life questionnaire and exacerbations

During the 52-week treatment period, lung function tests and QoL questionnaires were obtained every 3 months. Lung function measurements were performed according to the European Respiratory Society standard criteria [20]. The St George’s Respiratory Questionnaire (SGRQ) -a condition specific questionnaire- was used to measure health-related QOL and has been validated in bronchiectasis, with a minimal important difference of -4 [21]. An infectious exacerbation, before and during study treatment, was defined as an increase in respiratory symptoms, requiring antibiotic treatment [13]. Exacerbation frequency was reported on diary cards by the participants, documented by the treating physicians and double-checked through chart review by the principal researcher.

### Bronchiectasis severity index and radiological severity

The disease severity at baseline was calculated by using the bronchiectasis severity index (BSI) [2]. The BSI scoring system is a mortality prediction score and identifies individuals at risk of mortality, hospital admissions, and exacerbations. This scoring system has been developed and validated in multiple, large cohorts [2, 22]. At the time of the BAT trial [13] the BSI did not exist, therefore, in our analysis, the severity of the disease could only be calculated based on components of the BSI and not the total BSI score. However, these component scores of the BSI were also described as an independent predictor of the disease severity [2]. Beside the BSI, the radiological severity was calculated by using the bronchiectasis radiological indexed CT score (BRICS) [23]. The BRICS score is derived from combining the bronchial dilatation and the number of segments with emphysema on (high resolution) computed tomography (HRCT) and is validated in idiopathic and post-infective bronchiectasis**.**

### Statistical analysis

Statistical analysis was conducted by using IBM SPSS 25 for Windows. Descriptive statistics for patients treated with AZM or placebo were calculated at baseline. Discrete variables were presented as counts (percentage) and continuous variables as means with standard deviation (sd) if normally distributed and medians with interquartile range (IQR) if not normally distributed. Differences in the distribution of sputum markers and components of the severity index were compared using the Mann–Whitney U Test for two independent samples or Kruskal–Wallis test for comparisons of 3 or more. Differences in distribution of sputum markers at baseline and during an exacerbation were compared by using the Wilcoxon Signed Rank test for 2-related samples. The long-term effect of AZM on airway inflammation was compared to that of placebo using linear mixed model analyses. The adjusted associations between the inflammatory markers at baseline and treatment response, based on exacerbation frequency, lung function and quality of life, was shown in forest plots with 95% confidence interval (CI). A *p*-value < 0.05 was considered statistically significant.

## Results

### Baseline characteristics

A total of the 83 patients participated in the BAT trial and were included in this analysis. Baseline characteristics of the study population are shown in Table 1 according to the treatment group.

**Table 1:** Patient characteristics

	**AZM**	**Placebo**	*P*-value
**Total of patients**	43	40	
Age, mean (SD)	59.6 (12.3)	64.6 (9.1)	0.051
Female, n (%)	25 (63)	28 (65)	0.804
Aetiology of bronchiectasis, n (%)			0.850
Post infectious	15 (35)	13 (33)	
Idiopathic	12 (28)	15 (38)	
Asthma	7 (16)	7 (18)	
Auto-immune disease	3 (7)	2 (5)	
Common variable immune deficiency	1 (2)	1 (3)	
Primary ciliary dyskinesia	1 (2)	0	
Yellow nail syndrome	0	1 (3)	
Aspiration	1 (2)	0	
Mechanical obstruction	1 (2)	0	
Allergic bronchopulmonary aspergillosis	1 (2)	1 (3)	
Alpha-1-antitrypsin deficiency	1 (2)	0	
Baseline sputum microbiology, n (%)			
*Haemophilus influenzae*	13 (30)	9 (23)	0.617
*Pseudomonas aeruginosa*	6 (14)	6 (15)	0.855
Other(s)	24 (56)	25 (62)	0.874
No. Of exacerbations in year before study entry, median (IQR)	4 (2)	5 (3)	0.318
No. Of exacerbations during the study, median (IQR)	0 (1)	2 (2)	0.000
Pulmonary function tests, mean (SD)			
Δ (End-Start) FEV_1_, % pred	4.4 (9.1)	-0.9 (12.6)	0.046
Δ (End-Start) FVC, % pred	5.5 (14.0)	-2.6 (12.6)	0.013
Quality of life questionnaire’s, mean (SD)			
Δ (End-Start) Total score SGRQ	-11.3 (16.7)	-3.3 (15.8)	0.057

All values are expressed as mean (SD) or median (IQR) unless stated otherwise

*Abbreviations*: *AZM* Azithromycin, *FEV*_*1*_ Forced Expiratory Volume in one second, *FVC* Forced Vital Capacity, *IQR* Interquartile range, *SD* Standard deviation, *SGRQ* St George’s Respiratory Questionnaire

In total 399 sputa were obtained, eight of which were limited in volume and therefore 391 sputa could be analysed. An overview of the number of sputum samples per visit is shown in supplemental 1. From the 25 inflammatory markers analysed in each sample, a total of nine markers were excluded (Fractalkine, GMCSF, IFN-γ, IL-10, IL-12p70, IL-13, IL-17A, Il-4, IL-5) because no inflammatory activity was measured in these samples at baseline and during the study treatment (Table 2).

**Table 2:** Biomarkers in sputum specimen at baseline

	Overall *n* = 54	AZM *n* = 25	Placebo *n* = 29	*P*-value
ECP (ug)	3.75 (8.8)	3.8 (8.1)	3.6 (9)	0.828
Fractalkine (pg)	0 (0)	0 (0)	0 (0)	0.392
GCSF (pg)	0 (100.2)	0 (128.8)	0 (26.1)	0.307
GMCSF (pg)	0 (0)	0 (0)	0 (0)	1.000
GRO-α (pg)	14 (297.9)	13.9 (387)	17 (170)	0.668
IFN-γ (pg)	0 (0)	0 (0)	0 (0)	0.369
IL-10 (pg)	0 (0)	0 (0)	0 (0)	1.000
IL-12p70 (pg)	0 (0)	0 (0)	0 (0)	0.185
IL-13 (pg)	0 (0)	0 (0)	0 (0)	0.031
IL -17A (pg)	0 (0)	0 (0)	0 (0)	0.471
IL-1α (pg)	285.3 (871.7)	335.7 (660.3)	285.3 (1071.9)	0.869
IL-1β (ng)	6.5 (22.0)	6.4 (17.9)	7.5 (41.8)	0.735
IL-1RA (pg)	606.7 (1716.7)	549.9 (1993.2)	606.7 (1578)	0.842
IL-21 (pg)	7.61 (62)	3.1 (44)	8.4 (90)	0.503
IL-4 (pg)	0 (0)	0 (0)	0 (3)	0.737
IL-5 (pg)	0 (0)	0 (0)	0 (0)	0.879
IL-6 (pg)	5.5 (548.5)	0 (837.1)	63.9 (504.8)	0.623
IL-8 (ng)	22.1 (84.4)	25 (101.4)	22.1 (70.3)	0.381
IP-10 (pg)	53.5 (149.2)	42.6 (136.9)	76.3 (149.2)	0.695
MIP-1β (ng)	5.7 (9.7)	4.2 (6.5)	7.2 (14.4)	0.504
MIP-3α (pg)	0 (69)	0 (175)	0 (64)	0.496
MMP-9 (ng)	178.0 (329.7)	178 (316.9)	177.1 (375.1)	0.869
MPO (ug)	76.31 (240.4)	86.4 (178)	53.3 (270.2)	0.842
TNF-α (pg)	129.9 (1334)	238.4 (1019)	129.9 (1841)	0.854
VEGF (pg)	1665.3 (3794.6)	1221.3 (3789.3)	1700.1 (3985.2)	0.683

Baseline sputum inflammatory profile expressed per gram of sputum of study subjects. All values are expressed as median with inter quartile range (IQR); *p*-value: difference in inflammatory profile between the AZM treated patients and the placebo-treated patients at baseline

### Inflammatory profiles and the severity of bronchiectasis

At baseline, the severity of bronchiectasis was obtained using the following components of the BSI: age, FEV_1_% of predicted, exacerbation frequency, and bacterial colonisation [2]. A significant upregulation of pro-inflammatory markers was found in patients with a low FEV_1_ ( FEV_1_ < 50% of predicted) (ECP *p* = 0.021; TNF-α *p* = 0.007; IL-21 *p* = 0.041), and a higher number of exacerbations (≥ 3, ≥ 5, ≥ 7) in the year prior to the start of the study (MPO *p* = 0.004; ECP *p* = 0.003; IP-10 *p* = 0.011; VEGF *p* = 0.008; MMP-9 *p* = 0.041 (data not shown)). Surprisingly, patients colonized with *PA* showed no upregulation in inflammatory markers. Even a significant reduction was seen in this population as compared to non-*PA* for VEGF (*p* = 0.031), IL-8 (*p* = 0.01), and MMP-9 (*p* = 0.001) (Table 3). For patients colonised with *Haemophilus influenzae (HI),* a significant upregulation of pro-inflammatory markers was markedly found, expect for IL1-RA and GRO-α (Table 3). The inflammatory profile at baseline was not related to the component score ‘age’ of the BSI, and the radiological severity based on the BRICS (data not shown) [23].

**Table 3:** Baseline sputum inflammatory profile grouped according to *Pseudomonas aeruginosa* or *Haemophilus influenzae* colonization

	***Pseudomonas aeruginosa***			***Haemophilus influenzae***		
	**Yes** (*n* = 10)	**No** (*n* = 44)	*p*-value	**Yes** (*n* = 16)	**No** (*n* = 38)	*p*-value
**MPO (ug)**	87.5 (121.1)	75.9 (264.4)	0.373	249.9 (483.8)	49.5 (110.3)	**0.004**
**ECP (ug)**	3.0 (7.8)	4.3 (9.5)	0.449	7.7 (17.7)	2.6 (7.0)	**0.043**
**IP-10 (pg)**	19.3 (72.3)	70.2 (162.7)	0.180	77 (172.5)	43 (122.6)	0.133
**MIP-1β (ng)**	2.2 (6.4)	6.6 (10.9)	0.084	9.7 (32.2)	4.2 (8.2)	**0.022**
**VEGF (pg)**	567.9 (1327.7)	1719.5 (4025.6)	**0.031**	2261.7 (4656.8)	1332.7 (2840.7)	0.185
**TNF-α (pg)**	78.8 (157)	554.7 (1846)	0.120	1334.2 (4014)	37.1 (549)	**0.000**
**IL-1RA (pg)**	100.9 (2529.9)	640.0 (1579.0)	0.082	414.5 (920.3)	616.4 (2686.4)	0.405
**IL-21 (pg)**	0.28 (18)	16.3 (80)	0.215	44.1 (99)	0.0 (24)	**0.001**
**IL-8 (ng)**	5.6 (14.7)	38.4 (98.9)	**0.010**	82.3 (110.8)	17.4 (65.1)	**0.036**
**IL-1β (ng)**	5.1 (7.4)	7.5 (29.1)	0.161	30.5 (58.7)	3.7 (7.6)	**0.000**
**IL-6 (pg)**	0.0 (244.8)	38.4 (637.8)	0.341	6.5 (993.3)	2.8 (547.2)	0.935
**GCSF (pg)**	0.0 (18.4)	0.0 (129.9)	0.440	0.0 (218.1)	0.0 (67.2)	0.647
**GRO-α (pg)**	40 (177.9)	15.5 (418.4)	0.981	0.0 (129.6)	23.7 (463.1)	0.309
**IL-1α (pg)**	98 (410)	344.8 (945.4)	0.160	888.5 (1072.3)	176.3 (437.8)	**0.000**
**MIP-3α (pg)**	0.0 (20)	0.0 (118)	0.546	0.0 (1711)	0.0 (42)	0.309
**MMP-9 (ng)**	64.7 (68.8)	236.2 (455.1)	**0.001**	242.2 (608.3)	176.7 (306)	0.415

Data are presented as median with inter quartile range (IQR). *Pseudomonas aeruginosa* or *Haemophilus influenzae* colonisation at baseline (V1)

### The effect of AZM on the inflammatory profiles

**Fig. 1:**
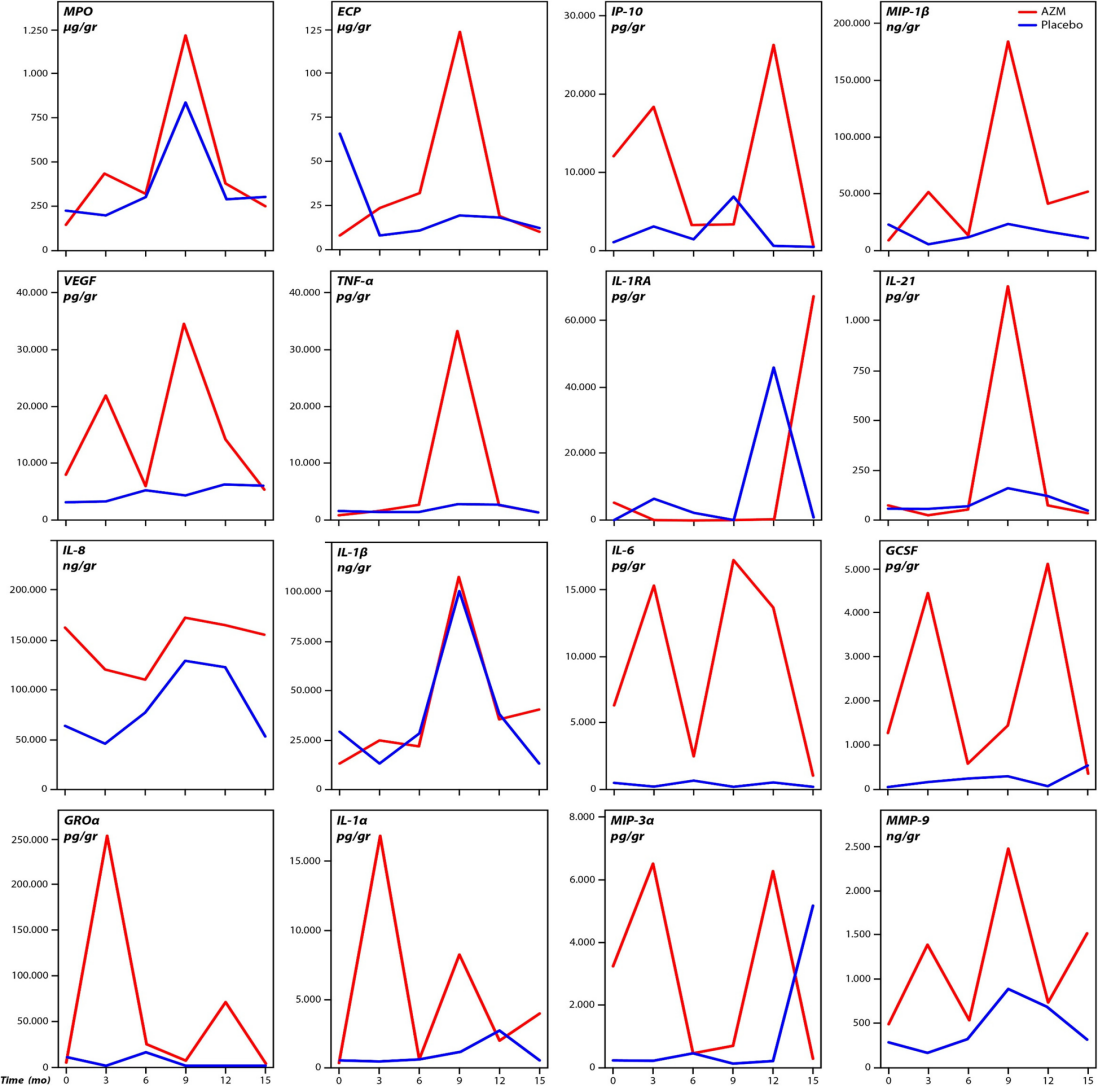
The effect of AZM maintenance treatment (per visit) on the inflammatory markers in sputum as compared to placebo

To verify the presumed immunomodulatory, anti-inflammatory effect of AZM over the time, stable-state sputum inflammatory markers from AZM-treated patients were compared to the placebo-treated patients using mixed models. An overview of the effect of AZM on the inflammatory markers during maintenance treatment per visit is shown in Fig. 1. No significant difference in pro-inflammatory cytokines were found in the AZM-treated patients as compared to the placebo-treated patients, and even higher levels of inflammation, except IL-1RA, were found during maintenance AZM treatment (supplemental 2).

### Sputum inflammatory profiles during an exacerbation

Out of the 117 exacerbations treated with antibiotics during the study, only 29 (25%) sputum samples were collected and analysed. Of these 29 sputum samples, nine (31%) patients were treated with AZM, and 20 (69%) patients were treated with placebo. In the total population (both AZM- and placebo-treated patients) a significant upregulation of the inflammatory profile was found during an exacerbation as compared to the baseline inflammatory profile. (MPO *p* = 0.003; ECP *p* = 0.004; MIP-1β *p* = 0.006; VEGF *p* = 0.027; TNF-α *p* = 0.005; IL-21 *p* = 0.010; IL-8 *p* = 0.013; IL-1β *p* = 0.003; IL-1α *p* = 0.005; MMP-9 *p* = 0.024). When this population is divided into AZM-treated patients and placebo-treated patients, the presence of an infectious exacerbation was reflected by an excessive and significant increase in inflammation especially in the placebo-treated patients (*p* = 0.012- *p* = 0.046) as compared to a non-significant upregulation of the inflammatory markers in the AZM-treated patients (Table 4).

**Table 4:** Sputum inflammatory profile in stable state and during an exacerbation grouped according to AZM treatment or placebo treatment

		**AZM**			**Placebo**	
	**Baseline** (*n* = 25)	**Exacerbation** (*n* = 9)	*p*-value	**Baseline** (*n* = 29)	**Exacerbation** (*n* = 20)	*p*-value
**MPO (ug)**	81.4 (203.1)	706.9 (1718.2)	0.068	53.3 (270.2)	222.3 (2260.1)	**0.015**
**ECP (ug)**	4.0 (9.0)	27.1 (151.2)	0.068	3.6 (9)	9.3 (67.8)	**0.015**
**IP-10 (pg)**	43 (167.6)	362.6 (1299)	0.144	76.3 (149.2)	108.4 (1270.7)	0.125
**MIP-1β (ng)**	4.3 (6.3)	54.8 (132.5)	0.144	7.1 (14.4)	19,688.2 (83,389.0)	**0.020**
**VEGF (pg)**	1443.3 (3936.9)	14,802.6 (29,729.1)	0.144	1700.1 (3985.2)	3990.8 (41,378.1)	0.078
**TNF-α (pg)**	175.7 (1025)	3629.5 (22,676.3)	0.068	129.9 (1841)	2287.0 (7732.4)	**0.020**
**IL-1RA (pg)**	617.2 (2112.2)	2760.9 (3649.9)	0.465	606.7 (1578)	1867.5 (17,356.4)	**0.020**
**IL-21 (pg)**	1.8 (47)	306.2 (680.1)	0.109	8.4 (90)	59.7 (1392.0)	**0.046**
**IL-8 (ng)**	25 (101.4)	148 (407.5)	0.068	22.1 (70.3)	80.9 (239.4)	0.053
**IL-1β (ng)**	6 (14.7)	72.8 (140)	0.068	7.5 (41.8)	39 (392.2)	**0.012**
**IL-6 (pg)**	0 (922.3)	290.5 (1705.2)	0.109	63.9 (504.8)	29.2 (809)	0.937
**GCSF (pg)**	0 (129.9)	0 (992.9)	0.180	0 (26.1)	0 (35.6)	0.173
**GRO-α (pg)**	9 (418.4)	155.8 (1753.3)	1.000	17 (170)	0 (289.7)	0.594
**IL-1α (pg)**	286.8 (717.4)	1829.7 (3282.2)	0.068	285.3 (1071.9)	1069.1 (3207.4)	**0.015**
**MIP-3α (pg)**	0 (236)	101 (1754.3)	0.180	0 (64)	67.6 (215.4)	0.463
**MMP-9 (ng)**	194.9 (328.5)	851.8 (2389.9)	0.144	177.1 (375.1)	337 (2311.3)	0.061

Data are presented as median with inter quartile range (IQR)

*Abbreviations*: *AZM* Azithromycin

### The relation between baseline inflammation and the effect of AZM maintenance treatment based on number of exacerbations, quality of life (QoL) and lung function

In this analysis the inflammatory markers at baseline were divided into values above the median and under the median to evaluate the relation between the levels of inflammation at baseline and the response on AZM maintenance treatment based on number of exacerbations, FEV_1_% of predicted and QoL.

#### Number of exacerbations

**Fig. 2:**
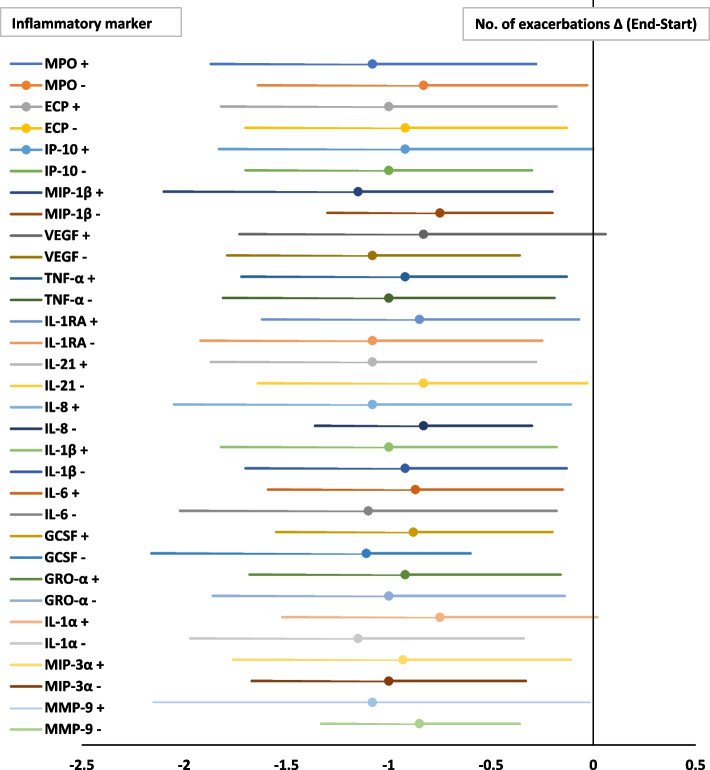
Association between baseline inflammatory profile and the effect of AZM maintenance treatment on the number of exacerbations in bronchiectasis. Forest plot; data are presented as Δ in number of exacerbations (End-Start) with 95% CI. X-axis: Decrease in number of exacerbations during 1-year AZM treatment. Y-axis: The + or – mentioned by the inflammatory markers represent respectively values above or below the median of the specific inflammatory marker at baseline

During AZM maintenance treatment a decrease in number of exacerbations was not related to a specific inflammatory marker at baseline. However, in patients with lower levels (under the median) of VEGF, IL1-RA, IL-6, GCSF, GRO-α, IL-1α at baseline a lower number of exacerbations during AZM maintenance treatment was found. These differences were marginal, and no specific inflammatory response is predictive for the effect of AZM maintenance treatment based on number of exacerbations. Figure 2 shows an overview of the inflammatory markers at baseline in relation to a decrease in number of exacerbations during maintenance AZM.

#### Quality of life

**Fig. 3:**
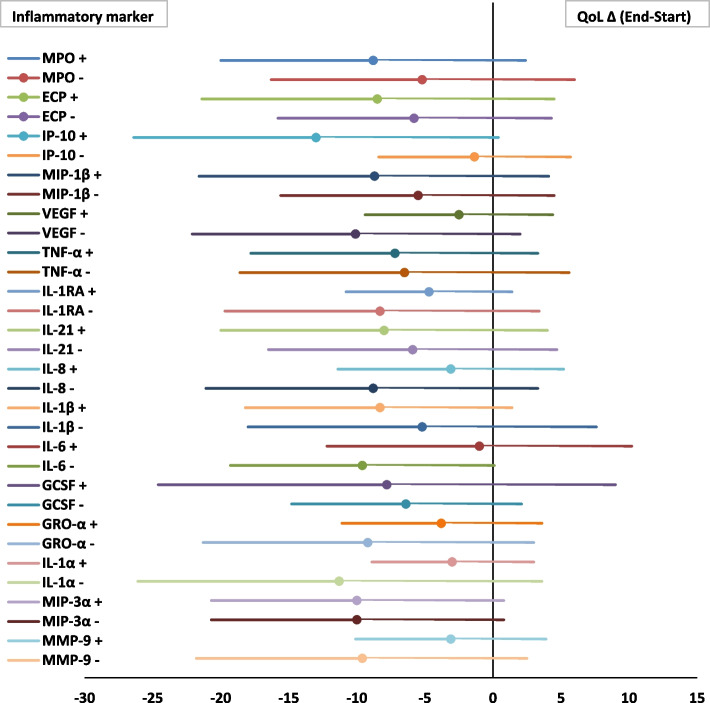
Association between baseline inflammatory profile and the effect of AZM maintenance treatment on SGRQ-total score in bronchiectasis. Forest plot; data are presented as Δ QoL (End-Start) with 95% CI. X-axis: Increase in QoL during 1-year AZM treatment based on a decrease of the SGRQ; St. George Respiratory Questionnaire. Minimal important difference of -4 correspondence with clinical relevance; Y-axis: The + or – represent respectively values above or below the median of the specific inflammatory marker at baseline

Figure 3 shows the relation between the inflammatory marker at baseline and the effect of maintenance AZM based on QoL by using the SGRQ. Overall, a decrease in the SGRQ total score was found during AZM maintenance treatment, representing a clinically relevant improvement of the QoL, expect for patients with, at baseline, higher levels of IL-8, IL-6, GRO-α, IL-1α, VEGF and lower levels of IP-10. When looking at distinct groups with higher or lower (compared to median) levels of inflammatory markers, however, no significant difference with respect to the effect of AZM treatment on QoL was found.

#### Lung function

**Fig. 4:**
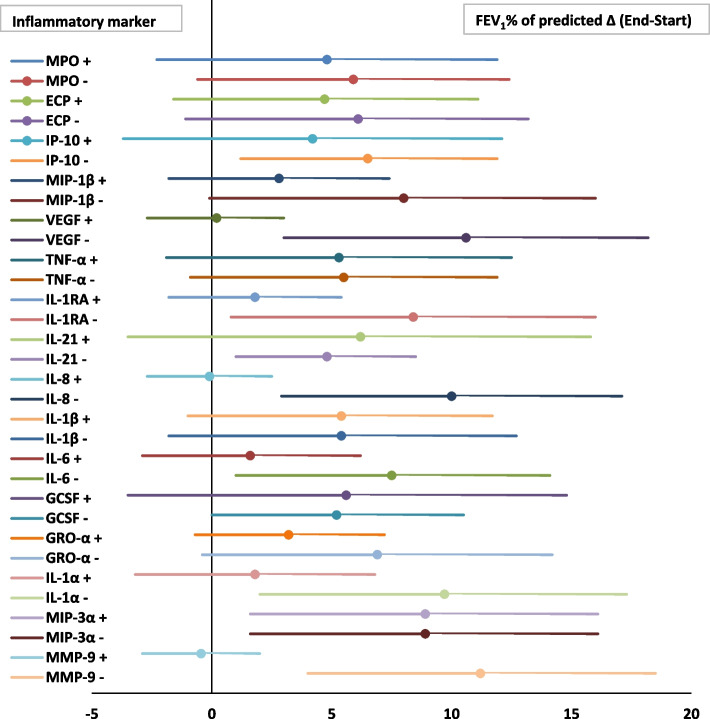
Association between baseline inflammatory profile and the effect of AZM maintenance treatment on change in lung function in bronchiectasis. Forest plot; data are presented as Δ FEV_1_% of predicted (End-Start) with 95% CI. X-axis: Increase in FEV_1_% of predicted during 1-year AZM treatment. Y-axis: The + or – represent respectively values above or below the median of the specific inflammatory marker at baseline

Figure 4 shows the relation between the inflammatory markers at baseline and the improvement of FEV_1_% of predicted during maintenance AZM treatment. A significant improvement in FEV_1_% of predicted was found for patients with lower levels of the inflammatory markers VEGF (*p* = 0.011) IL-8 (*p* = 0.019), IL1-α (*p* = 0.034) and MMP-9 (*p* = 0.03) at baseline as compared to the higher levels (above the median) of these inflammatory markers at baseline.

## Discussion

In the present investigator-initiated study we evaluated the inflammatory profile in expectorated sputum of patients with bronchiectasis participating in the BAT trial and treated with maintenance AZM or placebo for one year [13]. Our most remarkable finding was the fact that markers of airway inflammation remained stable or even increased during long-term macrolide treatment, suggesting that the clinically beneficial effect of macrolide treatment may not, or not as much, be driven by an anti-inflammatory effect as generally assumed. However, our results contrast with a recently published observational study whereby NETs were identified as a key marker of treatment response in bronchiectasis [12]. In this study, lower concentrations of NET proteins were found after one year of maintenance AZM, especially in patients with non-eosinophilic asthma and in patients with *PA*infection. NETs were also identified as a key marker of disease severity [12]. In our study, the inflammatory profile was examined at baseline in relation to the severity of the disease based on components of the BSI [2]. Similar results were found, whereby an increase in disease severity based on a FEV_1_ of < 50% predicted as well as an increase in exacerbation rate in the year prior to the start of maintenance treatment were reflected by an upregulation of the pro-inflammatory markers at baseline.

In contrast to previous studies, the patients colonized with *PA*in our analysis showed no inflammatory upregulation [2, 24, 25]. Moreover, levels of VEGF, IL-8 and MMP-9 were significant lower as compared to non-*PA* patients, probably due to the diversity of *PA*strains in bronchiectasis patients [26]. Another speculative hypothesis is that long-term colonization with *PA* would lead to a more chronic inflammation and, to a lesser extent, active inflammation. In our analysis, only 10 patients were colonized with *PA*, and in addition, only 16 patients with *HI*. Due to the small number of samples, the results must be interpreted by caution. Surprisingly, the presence of *HI* in our sputum samples at baseline showed a significant upregulation of the inflammatory markers, suggesting a more marked inflammation in these patients, with probably even more clinical signs of active inflammation**.** In our study, patients with *HI* had more exacerbations in the year prior to the study as compared to patients colonized with *PA.* Both in COPD and bronchiectasis patients, *HI*is related to an increase in inflammation with higher levels of IL-6, IL-8, IL-1β and MPO and is an independent predictor for future exacerbations [27, 28]. These higher levels of inflammation were also found in our analyses, as compared to both non-*HI* and *PA* colonization.

Our analysis showed that during an exacerbation the inflammatory response increased, with an excessive and significant increase in patients treated with placebo as compared to a non-significant increase in the AZM-treated patients. This may suggest that there is indeed a dampening effect on the inflammatory response with macrolide treatment, but exclusively during exacerbations and, at least in the current study not picked up during stable state. In addition, this finding of reduced upregulation of inflammatory markers during an exacerbation may be driving the clinical finding of a marked reduction in the number of exacerbations in the active treatment group during the BAT trial [13]. However, for this sub analysis the sample size was low, with a total of 29 sputum cultures collected during the exacerbation.

Prior to this study, we hypothesized that the inflammatory profile at baseline might be predictive of the effect of macrolide treatment, with higher levels of inflammation predicting better outcome, because of its supposed anti-inflammatory mode of action. However, in the current study, upregulation of no specific inflammatory marker at baseline was predictive for the treatment effect of AZM on exacerbations, FEV_1_% of predicted or QoL based on the SGRQ. Instead, lower values of (neutrophilic) inflammation expressed as based on VEGF, IL-8, IL-1α and MMP-9 were significantly associated with an increased improvement in FEV_1_% of predicted during AZM maintenance treatment. This may reflect reduced disease severity at baseline with a higher tendency to regenerate and improve during treatment. However, an association between downregulation of these specific inflammatory markers was not for other outcome measures such as exacerbation frequency and QoL, so the importance of this finding remains unsure.

Contrary to what is generally believed, the current study failed to show an attenuation of the inflammatory response in bronchiectasis patients with AZM treatment. A previous published systematic review of Zimmermann et al. [18] described an overall decrease in inflammatory markers in both sputum and serum samples of patients with various kinds of respiratory tract infections/inflammation, and skin and eye infections treated with macrolides. However, AZM treatment was more frequently associated with no influence on the immunological markers as compared to the other macrolides. In contrast to these results, a review of Huckle et al. [29] included 12 RCT’s of patients with stable COPD and described that prophylactic use of AZM (as compared to non-macrolides) is of benefit in reducing exacerbation frequency with reduced levels of a wide range of inflammatory markers in sputum. The effect of macrolide maintenance treatment on the inflammatory markers in the heterogeneous group of bronchiectasis patients has been studied in detail in a limited number of previous studies. Conflicting results were found, with a decrease of concentrations of IL-8, NE, and MMP-9 after treatment with clarithromycin (for 3 months) or roxithromycin (for 6 months) in two small open label studies [30, 31]. And in addition, one cohort study in patients with bronchiectasis and *PA*infection treated with maintenance AZM for one year found an decrease in NET concentrations [12]. However, in one other RCT included 20 patients, treatment with low- dose erythromycin for 2 months did not effected inflammatory markers as IL-1α, IL-8, and TNF-α [32]. Difference in treatment doses and duration of maintenance treatment of macrolides could be an explanation for these discordant results. Additionally, although we have measured a wide array of inflammatory markers, the effect of macrolide treatment on the immune system shows high complexity and is not fully understood yet. Therefore, one could argue that some effects may have been missed due underrepresentation of certain types of markers. However, this appears not very likely when considering the extensive panel, with markers representing different immunomodulatory pathways [18]. In light of the above; other factors likely contribute to the observed clinical benefit of macrolides treatment in bronchiectasis. A previous study showed that AZM attenuated IL-8 without attenuating neutrophilic inflammation, which is suggestive for the inflammatory response due to viral infections too [33].

This is to our knowledge the first study investigating the effect of macrolide treatment on airway inflammation during exacerbations. Interestingly, during an exacerbation AZM treatment appeared to have a dampening effect on the upregulated inflammatory response during an exacerbation, as compared to placebo. However, due to the small number of samples collected during an exacerbation, these results should be interpreted by caution. Nevertheless, this agrees very well with the most important clinical effect of long-term macrolide treatment, which is a marked reduction of the exacerbation frequency [13–15].

In our study the inflammatory profile was related to the severity of the disease expressed as an increase in number of exacerbations and a FEV_1_< 50% of predicted. This is in concordance with previous studies, mentioning upregulation of inflammatory markers (IL-8, IL-13, and NET proteins) as correlated to functional measurements of disease severity and an increase in exacerbations [8, 9, 12]. In our study mainly neutrophilic inflammation was found with a significant upregulation of MPO, ECP, IP-10, TNF-α, IL-21,VEGF, MMP-9 in relation to the severity of the disease, however higher levels of ECP suggested also an eosinophilic component. These results are in line with earlier findings suggesting that in bronchiectasis patients the inflammatory profiles are dominated by neutrophilic inflammation, but there is also a role for eosinophilic inflammation, and other innate immune mediators [4, 10, 12].

The results of our study are derived from a post-hoc analysis of the BAT trial [13] and showed additional information about sputum inflammation in bronchiectasis. However, there are some limitations to mention. From the total 83 patients conducted the BAT trial [13], a total of 391 sputa were collected during the study, from a maximum of 60 patients per visit (73%). During AZM treatment, the availability of sputum samples gradually decreased in contrast to the placebo-treated patients. Only the patients with persistent respiratory symptoms and probably a reduced response on AZM could still expectorate sputum at the end of the study. Second, mainly for logistical reasons, only a small number of sputum samples were collected during an exacerbation. And therefore, our subgroup analysis of the inflammatory profile during an exacerbation in the AZM-treated patients as compared to the placebo-treated patients must be interpreted carefully. In addition, the inflammatory markers were measured in spontaneously expectorated sputum, and not in bronchial alveolar lavage fluid, causing possible oral contamination and represent not exactly the inflammatory profile of the lower respiratory tract.

In conclusion, in this study, we investigated the longitudinal effect of macrolide maintenance treatment on airway inflammation in bronchiectasis patients during stable state disease and exacerbations and inflammation in relation to disease severity. We found that disease severity was related to a higher mainly neutrophilic inflammatory response at baseline, with a significant upregulation in patients especially colonized by *HI* and not in patients with *PA*. Maintenance AZM treatment did not attenuate the inflammatory response as compared to placebo, but a dampening effect on the immune response during exacerbation was seen in AZM-treated patients, which may be responsible for the observed clinical benefit of macrolide maintenance treatment.

For a better understanding of the pathways through which macrolides exert their effect in bronchiectasis, more research is needed. This may need to be more aimed at understanding an anti-bacterial or anti-viral effect and type of anti-inflammatory effect. Also, our observation of reduced upregulation of the inflammatory response during an exacerbation warrants further research.

## Supplementary Information

**Additional file 1: Supplemental 1.** Number of samples per visit in the total population. **Supplemental 2. **The effect of maintenance AZM on the inflammatory profile in sputum. Results after mixed model analysis. **Supplemental material 3. **Assays and sputum analysis.

## Data Availability

The datasets used and/or analyzed during the current study available from the corresponding author on reasonable request.
