# Supplementary material for: The effect of azithromycin on sputum inflammatory markers in bronchiectasis
Source: BMC Pulm Med. 2023 Apr 29;23:151. doi: 10.1186/s12890-023-02444-1 (PMC10148509; doi:10.1186/s12890-023-02444-1)
Supplement: Supplementary file 1 — Additional file 1: Supplemental 1. Number of samples per visit in the total population. Supplemental 2. The effect of maintenance AZM on the inflammatory profile in sputum. Results after mixed model analysis. Supplemental material 3. Assays and sputum analysis. [file 12890_2023_2444_MOESM1_ESM.docx]

**SUPPLEMENTAL MATERIAL**

**Supplemental 1. Number of samples per visit in the total population**

|  | **V1**  **(Start study)** | **V2**  **(3 months)** | **V3**  **(6 months)** | **V4**  **(9 months)** | **V5**  **(End of study)** | **V6**  **(Run out)** |
| --- | --- | --- | --- | --- | --- | --- |
| For all the inflammatory markers | 54 (65) | 50 (60) | 60 (72) | 56 (67) | 46 (55) | 47 (57) |
| AZM | 25 (46) | 25 (50) | 32 (53) | 29 (52) | 24 (52) | 23 (49) |
| Placebo | 29 (54) | 25 (50 | 28 (47) | 27 (48) | 22 (48) | 24 (51) |

Data are presented as numbers with percentages of the total population. At every visit, during stable state, all inflammatory markers were obtained and analysed.

**Supplemental 2. The effect of maintenance AZM on the inflammatory profile in sputum. Results after mixed model analysis**

|  | **V1^#^**  **Baseline** | **V2***  **3 months** | **V3***  **6 months** | **V4***  **9 months** | **V5***  **End of treatment** | **V6***  **Run-out** |
| --- | --- | --- | --- | --- | --- | --- |
| IL-8  ∆ (AZM-placebo)  *p-value* | 98314  0.267 | 51230  0.565 | 36878  0.650 | 16875  0.832 | 228435  0.011 | 102297  0.255 |
| IL-6  ∆ (AZM-placebo)  *p-value* | 5684  0.280 | 9770  0.270 | 14125  0.081 | -667  0.933 | 18183  0.043 | -2633  0.771 |
| GCSF  ∆ (AZM-placebo)  *p-value* | 1176  0.140 | 3613  0.02 | -115  0.934 | -375  0.784 | 1543  0.330 | -1297  0.408 |
| MMP-9  ∆ (AZM-placebo)  *p-value* | 189  0.322 | 101  0.931 | 1620  0.128 | 217  0.835 | 903  0.432 | 1235  0.300 |
| TNF-α  ∆ (AZM-placebo)  *p-value* | -697  0.224 | 2174  0.884 | 33037  0.016 | 3542  0.792 | 4076  0.787 | 1410  0.926 |
| IL-1β  ∆ (AZM-placebo)  *p-value* | -15885  0.130 | 414  0.995 | 10837  0.852 | 152  0.998 | 33078  0.603 | 36550  0.577 |

|  | **V1^#^**  **Baseline** | **V2***  **3 months** | **V3***  **6 months** | **V4***  **9 months** | **V5***  **End of treatment** | **V6***  **Run-out** |
| --- | --- | --- | --- | --- | --- | --- |
| MPO  ∆ (AZM-placebo)  *p-value* | -82  0.297 | 147  0.785 | 407  0.406 | 47  0.923 | 700  0.197 | 32  0.953 |
| ECP  ∆ (AZM-placebo)  *p-value* | -60  0.351 | 3.9  0.928 | 107  0.007 | 21  0.590 | 88  0.047 | 2.3  0.958 |
| IP-10  ∆ (AZM-placebo)  *p-value* | 1088  0.327 | 21409  0.062 | -7726  0.459 | 1638  0.873 | 230  0.984 | 6.5  1.000 |
| MIP-1β  ∆ (AZM-placebo)  *p-value* | -14558  0.196 | 25018  0.724 | 161912  0.013 | 1591  0.980 | 66143  0.353 | 44325  0.541 |
| VEGF  ∆ (AZM-placebo)  *p-value* | 4675  0.256 | 6402  0.605 | 28999  0.011 | 256  0.982 | 16117  0.196 | -1272  0.920 |
| IL-1RA  ∆ (AZM-placebo)  *p-value* | 5278007  0.195 | - | - | - | - | - |
| IL-21  ∆ (AZM-placebo)  *p-value* | 17  0.696 | -52  0.979 | 1018  0.045 | -17  0.973 | 5.2  0.993 | 16  0.987 |
| GRO-α  ∆ (AZM-placebo)  *p-value* | -7733  0.483 | 69619  0.308 | 4226  0.946 | 12487  0.839 | 162664  0.025 | -846  0.990 |
| IL-1α  ∆ (AZM-placebo)  *p-value* | 179  0.581 | -484  0.948 | 7271  0.282 | 339  0.959 | 1226  0.866 | 2242  0.771 |
| MIP-3α  ∆ (AZM-placebo)  *p-value* | 2973  0.159 | 4214  0.165 | -1337  0.628 | -1556  0.567 | -1836  0.557 | -6992  0.025 |

Data are presented as ∆ AZM-Placebo. ^#^results after independent samples T-test; *results after mixed model analysis with correction for baseline

**Supplemental material 3.**

**Assays**

The following assays were performed on sputum supernatants: ECP was measured using ECP monoclonal capture antibody (clone 614, Diagnostics Development, Uppsala, Sweden), ECP standard (ImmunoCAP ECP Calibrator Nieuwegein, the Netherlands), and biotinylated polyclonal detection antibody (Diagnostics Development, Uppsala Sweden) as described elsewhere.^35^

MPO was measured using duoset reagents DY3174 (R&D) and all steps were performed as described elsewhere.^36^

The following cyto-and chemokines were measured using eBioscience reagents: IP-10/CXCL10, MIP-1/CCL4, MIP-3/CCL20, MMP9, VEGF-A, INF-, TNF-, IL-1RA, IL-21, G-CSF, GM-CSF and GRO-/CXCL1 according to the manufacturer’s instructions. The plates were read on a Bioplex 200 (BioRad).

**Sputum analysis by UPLC-MS/MS**

^2^H_4_-succinic acid was added as internal standard to 100ul sputum. After vortexing, the sample was deproteinated using a 30 kD Amicon filter and 1 uL formic acid was added to the filtrate. 10uL of extract was injected to an UPLC-MS/MS (XEVO TQ-S micro, Waters, Milford, Massachusetts, USA) operated in negative ESI mode using MRMs for the preselected analytics. Chromatographic separation was chieved using an Acquity HSS T3, 200*2.1, 1.7uM analytical column and a linear gradient between solution B (methanol) and solution A (0.05M formic acid) with a flow rate of 0.5mL/min. The gradient was programmed: 0-3 min 95% A and 5% B, 3-3.1 min 95% A and 0% A and 5% B 100% B, 3.1 5 min 0% A and 100% B, 5-5.1 min 0% A 95% A and 100% B 5% B; all steps were linear. Data processing was performed using Masslynx 4.2 sofware.
